# Population density constrains coexistence of cooperative and cheating *Kluyveromyces lactis* through frequency-dependent physical interactions

**DOI:** 10.1371/journal.pone.0359779

**Published:** 2026-10-01

**Authors:** Rodrigo Zorrilla González, Michael Travisano

**Affiliations:** 1 Ecology, Evolution and Behavior, University of Minnesota, St. Paul, Minnesota, United States of America; 2 Bravo Farms and Laboratories, Los Saucos, Estado de México, Mexico; King Abdulaziz University, SAUDI ARABIA

## Abstract

Coexistence between cooperative and cheating individuals remains a central puzzle in social evolution. Here, we investigate how population density modulates coexistence using *Kluyveromyces lactis* strains that interact via physical attachment. Snowflake (cooperative) strains flocculate into multicellular clusters that settle efficiently, while Co-Uni (cheater) strains remain planktonic unless attached to Snowflakes, enabling them to exploit settling benefits without contributing to cluster formation. We manipulated settling density over 15 transfers (~60 generations) to alter encounter rates between strains while holding initial frequencies constant. At high densities, Co-Uni persistence was stabilized by frequent co-settling with Snowflakes; at low densities, reduced interaction frequency drove more rapid exclusion. Model-based projections revealed that both the rate of exclusion and the predicted equilibrium frequency of Co-Uni depend on settling density. Direct measurements of settling fitness confirmed that Co-Uni strains were less enriched in the settled fraction at low densities. These results demonstrate that interaction frequency, as modified by population density, can shape the ecological stability of social traits even in the absence of evolved enforcement or metabolic exchange. Our findings support the idea that density acts as a generalizable structural constraint on coexistence in microbial systems.

## Introduction

The strength and direction of ecological interactions can shift with environmental context, population structure, and evolutionary history [1,2]. Understanding how eco-evolutionary feedbacks influence the coexistence of cooperative and cheating behaviors remains a central challenge in evolutionary biology [3]. Individuals that gain disproportionate benefits from social interactions relative to their investment are often referred to as “cheaters” [4,5]. Examples of cheating include defection in group defense by capuchin monkeys [6] and group predation by lions [7]. Cooperation, by contrast, involves individuals incurring a cost to benefit others, as in reciprocal food-sharing among vampire bats [8] and alarm-calling in ground squirrels [9]. While theoretical work predicts that density and encounter rate can modulate the outcomes of social interactions [10,11], empirical tests that isolate these variables remain rare, particularly for facultative associations in which interactions are neither obligate nor metabolically costly [12]. Prior microbial studies have demonstrated density-dependent selection using metabolic public goods [13] and established ecological factors such as nutrient availability as indirect modulators of cooperation by influencing population density [14]. Here, we specifically isolate the role of direct physical interaction frequency in shaping cooperator–cheater dynamics by experimentally controlling population densities, thereby minimizing confounding effects from differential growth rates or changes in metabolic interactions.

Complex networks of interacting species characterize all communities. Understanding how ecological changes affect community composition and function is crucial for conservation in threatened environments [15]. While ecological experiments can reveal the impact of environmental changes, their scope and complexity often limit them. Microbes’ small size and rapid generation time offer unique opportunities to study evolutionary processes in real time, providing insights into the ecological mechanisms driving species interactions and co-existence [16–18]. Coexistence, however, is not the default outcome of such interactions: competing populations frequently undergo competitive exclusion, in which one strain displaces another over time, rather than stably persisting together.

In microbial communities, social and group interactions can shape, and be shaped by, ecological dynamics such as density, mixing, and spatial structure [19,20]. Most studies of microbial interactions involve metabolic products that create public goods, such as invertase or siderophore secretion [21,22], in which a costly, diffusible product provides a fitness benefit. Spatial structure and some degree of public good privatization can lead to coexistence of cooperators and cheaters [23]. Direct physical interactions have been studied in a few microbial species [24–27] in which the interactions are obligate. Here, we investigate a facultative, direct cell–cell contact association between cooperative and cheating strains of the yeast *Kluyveromyces lactis*. Throughout the experiment, we imposed daily “settling selection” by transferring only the bottom fraction of each culture after a brief gravity-settling period, thereby continuously favoring lineages that settle rapidly. In this system, multicellular flocculating “Snowflake” clusters settle efficiently, whereas unicellular, non-flocculating “Co-Uni” cells remain planktonic unless attached to Snowflakes [26]. This attachment enables Co-Uni cells to gain the benefits of rapid settling without paying the costs associated with flocculation.

The cost and benefit are well characterized in the ancestral selection experiment from which these strains were derived [26]: Snowflake clusters show significantly reduced maximum growth rate relative to unicellular strains (the cost of flocculation) while settling roughly 4-5x faster under settling selection (the benefit). This benefit is strain-specific rather than a general public good; Co-Uni cells gain settling fitness only when co-settling with clusters of conspecific Snowflakes. This system arose through de novo experimental evolution [26] of the snowflake genotype arising from a loss-of-function mutation (LOF) in the Ace2 locus, and the Co-Uni does not have this mutation [27]. Settling selection here should be understood as an experimental proxy for natural processes that favor aggregation, such as size-dependent predation, resource access and dispersal limitation, rather than a documented natural phenomenon in this species. The interaction is facultative, and asymmetrical: Snowflakes gain no detectable benefit from Co-Unis, while Co-Unis persist longer when Snowflakes are common, and encounter rates are high due to their higher growth rate [26]. Unlike metabolically cooperative systems, this interaction is governed entirely by physical co-sedimentation.

We manipulated settling density across four relative dilutions (1.0, 0.75, 0.5, and 0.25) to test whether population density modulates the stability of coexistence between Snowflake and Co-Uni strains. Both strains grow independently but differ in growth rate and settling dynamics. By serially transferring settled fractions and tracking strain frequencies over 15 transfers, we examined how interaction frequency, as modulated by population density, shapes the trajectory of cooperator–cheater dynamics and the model-predicted equilibrium frequencies that emerge from those trajectories. We hypothesized that reduced density would lower Co-Uni encounter rates thereby weaken the stabilizing effect of Snowflake association, leading to reduced Co-Uni persistence. Our 15-transfer experimental timeframe is comparable to the observed timescale of adaptive change in this system (emergence from the unicellular ancestral state within 10 transfers; [26,28]), allowing us to test whether similarly rapid evolutionary changes would recur within our own experiment.

This system provides a minimal, well-controlled platform for studying social trait dynamics in microbial populations. Its simplicity, physical interaction without metabolic exchange, facultative cheating, and independently viable strains, make it a broadly useful platform for testing how ecological context shapes cooperation, exploitation, and coexistence. While this study focuses on population density as a modulator of interaction frequency, the system is also well-suited to investigating other key environmental contexts that affect social evolution, such as nutrient limitation and resource partitioning. Our findings support a broader view in which population density functions not just as a demographic parameter but as a structural determinant of social evolution, shaping whether interaction-dependent traits are stabilized, lost, or exploited.

## Methods

### Strains and growth environment

We used two derivatives of the *Kluyveromyces lactis* strain NRRL Y-1140 (ATCC 8585), originally selected for rapid settling in non-agitated liquid media by Driscoll & Travisano (2017). Both strains have been sequenced [27]. The first strain, Y8sn-1, exhibits a flocculent multicellular phenotype and is hereafter referred to as “Snowflake.” Single-colony sequencing showed that the Snowflake genotype contains a frameshift mutation in the Ace2 locus (A -> AT), generating a stop codon and a partial or full loss of function, as did almost all other snowflake genotypes from the original selection experiment (see [27]). A genetically constructed knockout of the Ace2 locus in the ancestral strain generated the snowflake phenotype [27], as do Ace2 LOF mutations in *Saccharomyces cerevisiae* [29–31]. The second strain, Y8U, is a co-evolved unicellular lineage that co-settles with Snowflakes via physical attachment and is referred to as “Co-Uni.” It does not have a mutation in the Ace2 locus, compared to its ancestor. Snowflake Y8sn-1 displays a multicellular-like phenotype, forming dense clusters that settle rapidly under gravity. Co-Uni Y8U acts as a cheater: it does not flocculate independently but attaches to Snowflake clusters to benefit from their settling dynamics, while maintaining a higher intrinsic growth rate.

All strains were grown in 5 mL YPD media (1% yeast extract, 2% peptone, 2% dextrose) at 30°C with shaking at 250 rpm in 25 mm × 150 mm glass culture vials.

### Settling selection protocol

To select for settling ability, five replicate cocultures were initiated by inoculating 50 μL each of overnight Snowflake and Co-Uni precultures into sterile YPD. Each day, cultures were vortexed vigorously for 5 seconds to fully resuspend biomass. We then transferred 1.5 mL of culture into microcentrifuge tubes, allowed them to settle undisturbed at room temperature for 7 minutes, and pipetted 100 μL from the bottom of each tube into fresh 5 mL YPD [26]. This procedure selectively enriches for strains that sediment more rapidly. Populations were propagated under this settling regime for 15 consecutive daily transfers. The 15-day timeframe was chosen to match the known emergence timescale of these phenotypes: *K. lactis* Snowflakes emerged rapidly (within 10 transfers) in the original experiment [26,28], which also included the appearance and rise to substantial frequency of the snowflake phenotype. We used a comparable window to test whether similarly rapid evolutionary changes would recur, scanning for changes in morphology and phenotype frequencies over 15 transfers. Under these experimental conditions, new adaptive changes are unlikely to arise and fix within this duration [27]. Aliquots (500 μL) from transfers 0 and 15 were stored in 25% glycerol at –80°C for downstream analysis.

### Determination of colony forming units (CFU)

Population sizes of each strain were estimated every three days via a modified CFU spotting method designed to preserve Snowflake cluster integrity [26]. Cocultures were serially diluted in sterile YPD, and 100 μL of each dilution was gently spotted onto dry YPD agar plates. This approach minimizes mechanical disruption of Snowflake clusters, which can occur during traditional plating techniques involving spreading or wet agar surfaces.

Plates were incubated undisturbed at room temperature, which allowed early visual identification of multicellular Snowflake colonies before they expanded to the point of overlapping. Snowflake clusters were counted after 24 hours, when colonies were still distinct; Co-Uni colonies were counted after 48 hours to ensure reliable visibility. This timing maximized accuracy while maintaining consistent growth conditions across phenotypes.

CFU counts reflect functional reproductive units: for Snowflake, the intact cluster (the unit under selection during settling), and for Co-Uni, the individual cell. These were treated as the propagules contributing to the next generation. When relative abundances differed substantially, separate dilutions were used to reliably enumerate each morphotype. Five technical replicates per biological replicate were recorded.

### Assessing coexistence

As a follow-up to the main experiment, we tested whether Co-Uni persistence could be due to the appearance of new mutations and subsequent evolutionary changes. Cocultures were initiated with Co-Uni isolates from day 15 and Snowflake isolates from day 0 and compared with isolates of Co-Uni and Snowflakes from day 0. Five replicate populations were initiated for each treatment and transferred for six days under settling selection, with CFUs determined by spot plating. Growth curves were generated by measuring optical density at 600 nm using a Tecan Infinite 200 Pro microplate reader. Measurements were taken every 15 minutes using 96-well flat-bottom plates filled with 200 µL of media. Colonies were started with a 1:100 dilution inoculum, and samples from these populations were analyzed to calculate intrinsic growth rates.

### Determination of density-dependent effects on co-existence

To examine how population density during settling influences short-term strain dynamics, we established five replicate cocultures at each of three “relative settling density” treatments: 0.25, 0.50, and 0.75. Settling density was manipulated by diluting cultures with sterile YPD prior to selection each day, resulting in different concentrations of cells entering the 7-minute settling phase. Cultures were propagated for 15 daily transfers, matching the timeframe used in our primary coexistence experiment (see above), over which we found no evidence of substantial evolutionary change. Population sizes were tracked throughout the experiment, and samples from days 0 and 15 were cryopreserved in 25% glycerol at –80°C. One replicate from the 0.50 density treatment was excluded from analyses due to evidence of contamination. We fit three-parameter exponential decay models to strain frequency trajectories, using the asymptotic parameter as a heuristic projection of the long-term expectations under the observed ecological dynamics.

### Determination of relative fitness during sinking

To assess the effect of population density in settling ability, five biological replicates per coculture and settling group were prepared. Cocultures were created by combining equal volumetric parts of Snowflake and Co-Uni monocultures at their assigned settling densities and mixed thoroughly. To limit changes to population structure caused by destructive sampling, 1.5 mL microcentrifuge tubes were prepared for both “before” and “after” settling conditions. Samples before settling were taken from the middle of a 1.5 mL microcentrifuge tube, and samples after settling were selected from the bottom of a separate tube following 7 minutes of settling. CFUs were determined through spotting, and fitness was calculated as described below.

Relative fitness represents the increase in population size of the Co-Uni settled portion after 7 minutes of settling relative to the Snowflake settled portion. A higher relative fitness value indicates superior settling ability of Co-Uni compared to Snowflake.


$$\begin{array}{c} \text{Relative Fitness}=ln\left(\frac{{\text{CFU}}_{\text{Co}-\text{Uni},\text{ after settling}}}{{\text{CFU}}_{\text{Co}-\text{Uni},\text{ before settling}}}\right) \end{array}$$

### Statistical analysis

All statistical analyses were conducted using R version 4.4.1 [32]. All statistical analyses were conducted using R version 4.4.1. Statistical significance was assessed at α = 0.05 throughout. Mixed-effects linear regression models were implemented with the lme4 package [33], including fixed effects for treatment group and time point, and random intercepts for individual replicate populations. Significance of fixed effects was assessed using Type III ANOVA.

Intrinsic growth rates were estimated from growth curves using the growthcurver package [34], and analyzed via multifactorial ANOVA to test for treatment-level differences.

To evaluate the impact of population density on settling ability, we calculated relative fitness as the difference in logged CFU counts before and after settling. Linear regression models were used to test for density effects on fitness, with significance determined by ANOVA.

To evaluate strain-specific dynamics and assess projected equilibrium outcomes, we fit three-parameter exponential decay models with non-zero asymptotes to Co-Uni:Snowflake CFU ratios and Co-Uni CFU counts, and logistic growth models to Snowflake CFU counts. These models were selected to reflect the observed monotonic decline (Co-Uni) and saturating growth (Snowflake) trajectories over time and were fit to each biological replicate across all 15 daily transfers under the assumption that early ecological dynamics approximate equilibrium approach in the absence of substantial evolutionary change. From each nonlinear fit, we extracted the asymptotic parameter (c) as a projected equilibrium value and regressed these estimates against settling density to assess density dependence. We interpret these asymptotic values as descriptive summaries of short-term ecological trends, not mechanistic predictions of long-term equilibrium. Replicates for which nonlinear fits failed to converge were excluded from downstream analyses (One replicate at the density failed to converge for Snowflake logistic fits; all other replicates were retained; see S13 & S14 Tables in S1 File). In parallel, we log_10_-transformed Co-Uni:Snowflake ratios, Co-Uni and Snowflake CFU counts and fit linear models separately for each replicate to quantify exclusion slopes, which reflect the rate of competitive displacement over time. These slopes were similarly regressed against settling density to test for density effects. Time was treated as a continuous variable in days, and replicate-level R^2^ values for log-linear fits were calculated manually from residual and total sums of squares.

All data manipulation and plotting were performed using tidyverse [35]. Model outputs were summarized with broom [36] and broom.mixed [37].

## Results

### Co-Unis persist in coculture with Snowflakes

Driscoll & Travisano found that selection for rapid settling in the unicellular *K. lactis* strain NRL Y-1140 led to the multicellular “Snowflake” phenotype and the co-evolved unicellular “Co-Uni” phenotype within 10 transfers. To test whether similarly rapid evolutionary changes occurred in our experiment, we determined population densities by plating serial dilutions of five replicate vials every three days over 15 daily transfers.

Using a multiple linear regression model, we found significant effects of transfer number, phenotype, and their interaction on population density (all p < 0.001). The significant interaction between transfer number and phenotype (F_1,296_ = 186.13, p < 0.001) indicated differential changes in population densities between Snowflake and Co-Uni strains (S1 Table in S1 File). ANOVA confirmed the effects of phenotype (F_1,296_ = 8637.09, p < 0.001) and the interaction with transfer number (F_1,296_ = 182.50, p < 0.001), but transfer number alone was not significant (F_1,296_ = 1.09, p = 0.297) (S2 Table in S1 File).

Our results confirm that Snowflake and Co-Uni co-existence can be replicated. In coculture, Co-Uni population densities steadily declined, while Snowflake population densities increased reciprocally over the initial transfers (S1A Fig in S1 File).

To determine whether Co-Uni persistence was due to rapid adaptive changes, we compared population densities of isolates from the start (Day 0) and end (Day 15) of the experiment, cocultured with Snowflake isolates from the start of the experiment. A linear mixed-effects model revealed significant effects of transfer number (t_288_ = −12.87, p < 0.001), phenotype (t_288_ = −50.25, p < 0.001), and their interaction (t_288_ = 14.32, p < 0.001) (S3 Table in S1 File). Type III ANOVA confirmed the significance of transfer number (F_1,288_ = 37.84, p < 0.001), phenotype (F_1,288_ = 4875.04, p < 0.001), and their interaction (F_1,288_ = 371.18, p < 0.001) (S4 Table in S1 File). These results indicate that evolutionary changes over the 15-day experiment did not significantly affect coexistence dynamics (S1B Fig in S1 File). This is consistent with results from the original selection experiment, in which no systematic genetic changes were observed across ten independent replicates for the Snowflake genotypes [26,27].

Finally, growth rates for Snowflake and Co-Uni isolates from the start and end of the experiment were assessed using a two-way ANOVA. We found a significant effect of phenotype (F_1,52_ = 26.29, p < 0.001), indicating that Co-Uni growth rates were consistently higher than Snowflake growth rates. There was also a minor but significant effect of isolate day (F_1,52_ = 5.28, p = 0.026), but no significant interaction between phenotype and isolate day (F_1,52_ = 0.65, p = 0.425) (S5 Table, S2 Fig in S1 File).

### Density during settling influences coexistence dynamics

We manipulated initial population densities during settling (“relative settling density”) to test whether Co-Uni persistence was sensitive to Snowflake crowding and interaction opportunities. We hypothesized that lower densities would reduce Snowflake competition and limit Co-Uni’s ability to establish co-settling contact, potentially influencing coexistence outcomes on ecological time scales.

A linear mixed-effects model incorporating time, phenotype, and density as fixed effects showed significant effects of time (t_1169_ = −37.07, p < 0.001) and phenotype (t_1168_ = −50.09, p < 0.001) on population density. Settling density alone did not significantly affect population density (t_1168.0_ = −0.77, p = 0.444). Notably, interactions between time and phenotype (t_1168_ = 30.61, p < 0.001), and time and density (t_1168_ = 19.47, p < 0.001) were significant, indicating that population density changes are influenced by both phenotype and settling density. Additionally, a three-way interaction among time, phenotype, and density was significant (t_1168_ = −12.94, p < 0.001), suggesting complex interactions (S6 Table in S1 File).

A Type III ANOVA (S7 Table in S1 File) confirmed these findings, revealing significant effects of time (F_1,1169.4_ = 475.83, p < 0.001), phenotype (F_1,1167.9_ = 2508.83, p < 0.001), and density (F_1,1168_ = 5.15, p = 0.023), along with their two- and three-way interactions.

In summary, Co-Uni population densities consistently declined due to changes in settling density, and Snowflake populations were differentially affected (Fig 1).

**Fig 1:**
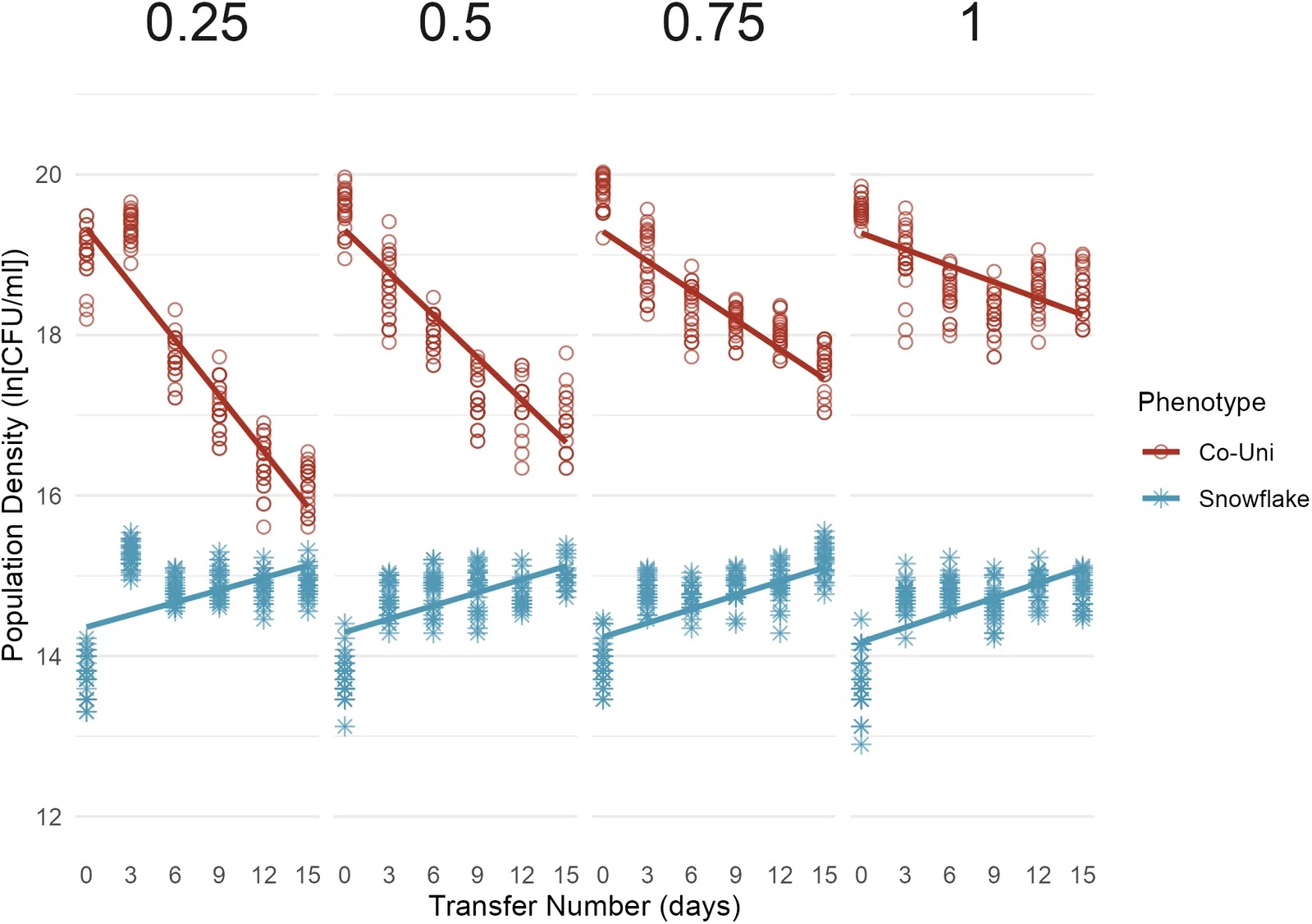
Low settling densities reduce Co-Uni population densities over time. Change in population density of Co-Uni and Snowflake strains across different relative settling densities over 15 daily transfers in coculture, shown as the natural logarithm of colony-forming units per milliliter (ln[CFU/mL]). Lines represent predicted population densities from a linear mixed-effects model fit to replicate data. Transfers were conducted every 24 hours under settling selection. The minimum detection threshold for both strains was approximately 12.5 ln(CFU/mL). Panel headers (0.25, 0.5, 0.75, 1) indicate relative settling density.

### Projected equilibrium frequencies depend on settling density

To evaluate how settling density affects coexistence dynamics between Snowflake and Co-Uni strains, we analyzed the trajectory of log-transformed CFU ratios over time for each biological replicate (Fig 2A). We fit a three-parameter exponential decay model of the form:

**Fig 2:**
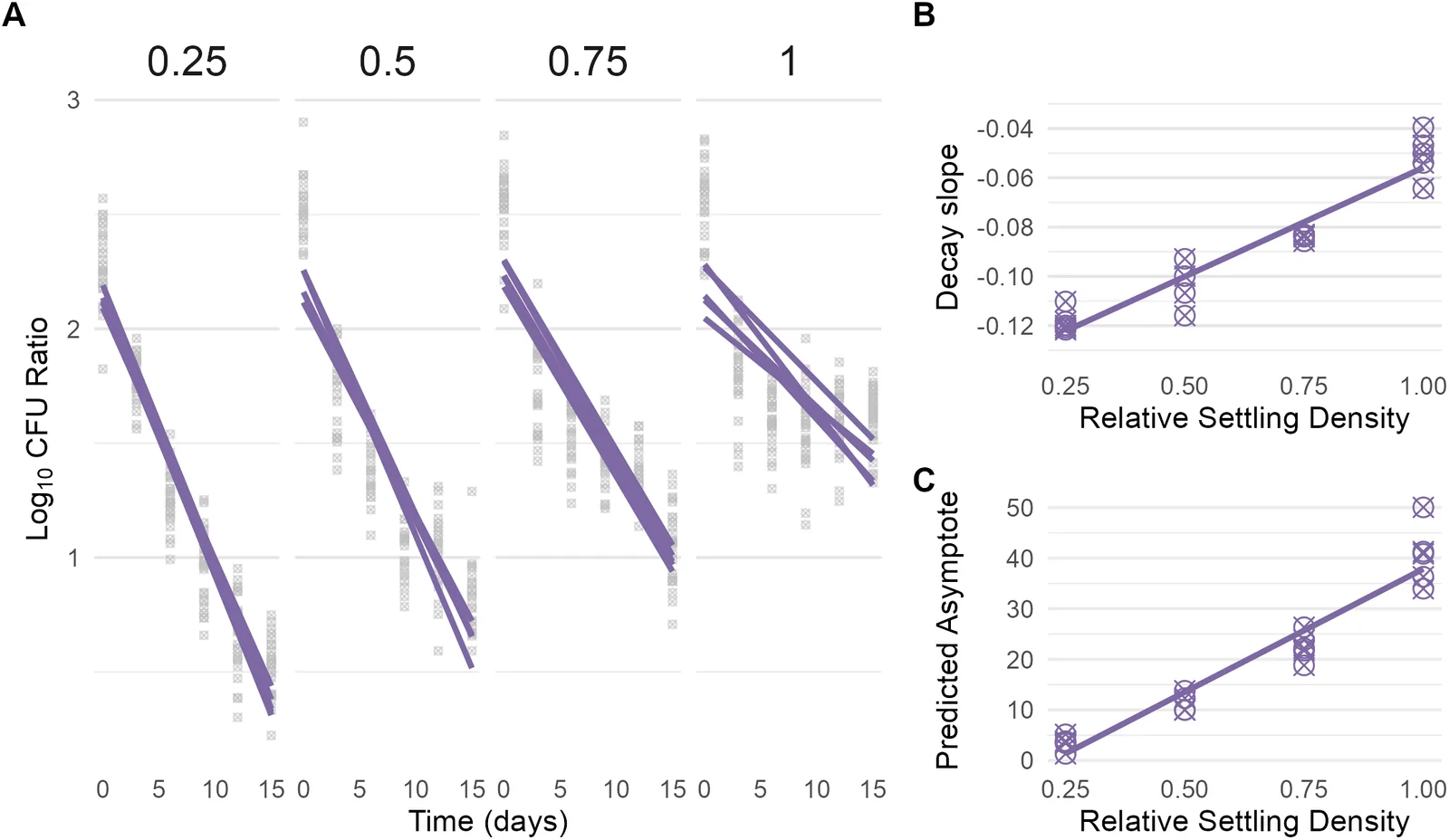
Settling density modulates the strength and endpoint of frequency-dependent exclusion. A) Log-transformed CFU ratios (Co-Uni/Snowflake) over time (days) for each replicate at each settling density; panel headers (0.25, 0.5, 0.75, 1) indicate relative settling density. Lines show replicate-level linear fits; grey points show raw data. B) Linear regression of replicate-level slope values (log_10_ CFU ratio/day) against settling density. Slopes quantify the rate of directional exclusion; more negative slopes indicate stronger Co-Uni decline. (C) Projected asymptotic CFU ratios estimated from nonlinear model fits. A significant positive relationship indicates that settling density shifts the expected strain ratio under ecological time scales.


$$\begin{array}{c} {log}_{\text{1}\text{0}}\left(\frac{\text{Co}-\text{Uni CFU}}{\text{Snowflake CFU}}\right)=a\times exp(b\times \text{time})+c \end{array}$$

where the slope parameter (b) provides an estimate of the net selection coefficient acting on Co-Uni strains under each settling density. Model fits were performed independently for each biological replicate. R^2^ calculations confirmed that log-linear fits consistently captured strain dynamics (S8 Table in S1 File).

Linear regression of slope values extracted from replicate-level log-linear models revealed a significant positive relationship between settling density and selection strength (t_17_ = 13.4, p < 0.001; S9 Table in S1 File), indicating slower relative decline of Co-Uni at higher densities (Fig 2B). This relationship was supported by ANOVA (F_1,17_ = 178.74, p < 0.001; S10 Table in S1 File). To estimate long-term ecological outcomes of these dynamics, we extracted asymptotic values from the same replicate-level model fits and analyzed their relationship with settling density (Fig 2C). A linear regression revealed a significant positive effect (t_17_ = 13.88, p < 0.001; S11 Table in S1 File), supported by ANOVA (F_1,17_ = 192.57, p < 0.001; S12 Table in S1 File). These results demonstrate that settling density modulates both the rate of exclusion and the projected equilibrium frequency between Co-Uni and Snowflake strains under ecological timescales.

To assess strain-specific dynamics directly, we modeled CFU trajectories separately for Co-Uni and Snowflake populations across all density treatments. Co-Uni abundance was well-described by an exponential decay model; fit parameters are summarized in S13 Table in S1 File. Snowflake abundance was fit using a logistic growth model, with parameters summarized in S14 Table in S1 File. This model converged successfully for 19 of 20 biological replicates. For Co-Uni, both the rate of decline and projected equilibrium CFU counts varied significantly with settling density (S15–S18 Tables; S3 Fig in S1 File). In contrast, Snowflake population trajectories remained comparatively stable across densities, with no significant changes in growth rates or equilibrium CFUs (S19–S22Tables; S4 Fig in S1 File). Snowflake CFU counts showed greater variation than Co-Uni CFU counts, likely reflecting a combination of variation in cluster age within the population and the sensitivity of intact clusters to mechanical disruption during plating (see Methods) [26]. These results confirm that density modulates Co-Uni co-existence dynamics but has little effect on Snowflake dynamics over this timescale.

### Decline in Co-Uni relative fitness at low settling densities is due to reduced co-settling ability

We tested whether the reduced persistence of Co-Uni at low densities was due to limited opportunities to attach to Snowflake clusters during settling. We first quantified relative fitness during settling as the log-ratio difference between settled and unsettled CFU counts for Co-Uni versus Snowflake strains (see Methods). Negative values indicate reduced enrichment of Co-Uni in the settled fraction relative to Snowflake.

Co-Uni relative fitness increased significantly with settling density (Fig 3A). Linear regression revealed a strong positive relationship (β = 0.531, p < 0.001; S23 Table in S1 File), and ANOVA confirmed a significant effect of density (F_1,18_ = 41.61, p < 0.001, S24 Table in S1 File).

**Fig 3:**
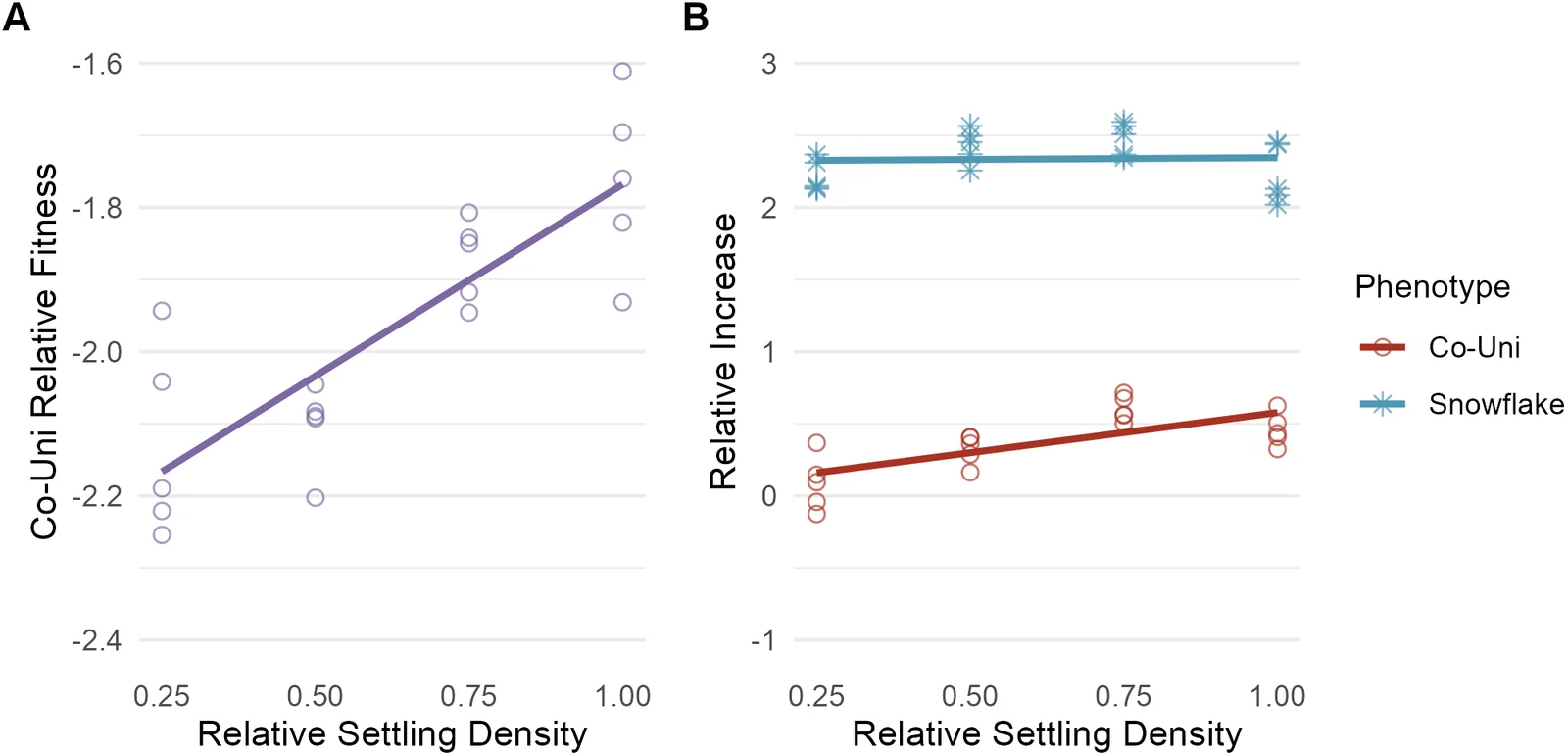
Co-Uni performance during settling improves at higher relative settling densities. A) Co-Uni relative fitness increases with settling density. Each point represents a biological replicate; the fitted line shows the result of a simple linear regression (R² = 0.6981, adjusted R² = 0.6813). B) Net change in Co-Uni and Snowflake strain abundance during settling, shown as ln(settled CFU / unsettled CFU). Points represent individual replicates; lines show linear fits across relative settling densities for each phenotype (Co-Uni: R^2^ = 0.9727, adjusted R^2^ = 0.9705). Only Co-Uni shows a positive slope, indicating density-dependent improvement in settling performance.

Next, we calculated the net change in abundance (“relative increase”) for each strain as ln(settled CFU / unsettled CFU) and examined how this value varied with settling density (Fig 3B). A linear model including phenotype and its interaction with density revealed a significant density-by-phenotype interaction (β = –0.531, p = 0.011; S25 Table in S1 File), confirmed by ANOVA (F_1,36_ = 7.23, p = 0.011; S26 Table in S1 File). Only Co-Uni showed a positive relationship between settling success and density; Snowflake performance remained stable across treatments. This pattern supports the hypothesis that Co-Uni performance is constrained at low density by reduced encounter rates with cooperative clusters, consistent with a density-modulated limitation on co-settling ability.

To identify which parameters best explained variation in Co-Uni settling fitness, we compared multiple candidate models and found that settling density alone provided the best fit (adj. R^2^ = 0.68, AIC = − 30.3; S27 Table in S1 File). In this model, higher density predicted significantly greater Co-Uni Enrichment in the settled fraction (β = 0.53, p < 0.001). Adding other covariates did not substantially improve explanatory power. Thus, population density is the principal ecological factor underlying variation in Co-Uni performance under settling selection in this system. Table 1 summarizes those density-dependent effects across the four key metrics examined in this study.

**Table 1:** Effects of settling density on Co-Uni-Snowflake coexistence dynamics.

Response variable	β (density)	SE	t	p	Source
Selection strength (Co-Uni:Snowflake decay slope)	0.088	0.007	13.4	<0.001	S9, S10
Asymptotic Co-Uni:Snowflake ratio	48.68	3.51	13.88	<0.001	S11, S12
Co-Uni equilibrium	3.13x10^8^	4.16x10^7^	7.54	<0.001	S15, S16
Relative settling fitness	0.531	0.082	6.45	<0.001	S23, S24

## Discussion

### Density-dependent stabilization of microbial co-existence

Experimental evolution studies have demonstrated stable co-existence between de novo evolved strains and unrelated species, yet competitive exclusion remains the dominant outcome [38–42]. This presents a paradox because co-existence is a defining characteristic of all ecosystems, and is expected when strong stabilizing and/or equalizing effects are present [1]. Research into the factors that influence these effects may provide insights into resolving this apparent paradox

This study examined the impact of density-dependent social interactions on the co-existence of two co-evolved *K. lactis* strains: “Snowflake” and “Co-Uni”. Snowflakes cooperatively settle through social flocculation, creating a public good exploited by Co-Unis via cellular attachment [26]. In the cooperator-cheater terms introduced above, Co-Uni is a cheater in the classical sense, acquiring the benefits of this costly cooperation without contributing to it. Our results show that this cheating strategy is not unconditionally successful: its viability depends on encounter rate with cooperators, breaking down when settling density is low, positioning population density as a previously underexplored axis of cheater control alongside more commonly studied mechanisms such as spatial structure and kin discrimination. While similar density-dependent dynamics have been shown in metabolic systems [13], our approach manipulated total density after full growth, controlling for growth-rate differences between strains. By manipulating population density during settling, we showed that Co-Uni persistence depends on interactions with Snowflakes. Our findings suggest that population density and interaction frequency influence the stabilizing effects of social interactions in microbial communities.

Snowflake clusters facilitate Co-Uni persistence by providing a physical structure that Co-Uni cells can attach to during settling. This cell-cell adhesion is specific, as Co-Unis do not adhere to themselves or multicellular *S. cerevisiae* [26,28]. This interaction creates a stabilizing mechanism; when Snowflake density is high enough to support frequent contact, Co-Uni settling ability increases despite its inability to form clusters independently. Co-Uni’s faster growth rate provides an equalizing force that mitigates its lower settling success. Together, these effects satisfy classical coexistence criteria: stabilizing mechanisms reduce the relative fitness advantage of one strain when rare, while equalizing mechanisms minimize average fitness disparities. As settling density declines, interaction frequency drops, weakening the stabilizing effect of Snowflake aggregation. Our model projections show that under low relative settling densities, Co-Uni fails to persist. These projections are based on empirical model fits and are intended as heuristic projections of short-term ecological dynamics, rather than mechanistically predicted equilibria. These results support the conclusion that co-existence in this system is primarily shaped by the frequency of stabilizing social interactions. Similar experiments with non-flocculant *S. cerevisiae* resulted in exclusively Snowflake populations, supporting the role of social flocculation in maintaining co-existence [43]. Likewise, cocultures of non-flocculant Snowflake *S. cerevisiae* and flocculant unicellular *S. cerevisiae* result in competitive exclusion by the Snowflakes [44].

### Game-theoretic perspective on density-modulated exploitation

The interaction between Snowflake and Co-Uni cells can be understood through the lens of a Snowdrift game, a scenario in which cooperation is favored when rare but exploited when common [10,21]. Snowflake clusters generate a public good—settling capacity—that Co-Uni cells can exploit through attachment, without incurring the cost of cluster formation. In such systems, the success of non-cooperators depends on the frequency of cooperators, and the cost-benefit ratio of cooperation shapes stable trait mixtures [13,22,45,46]. Our findings extend this framework by showing that population density modulates these dynamics, not just frequency. Here, density refers to the absolute concentration of cells, whereas frequency refers to the relative proportion of Co-Uni to Snowflake within the population; the two can vary independently. At high densities, frequent interactions allow Co-Uni to exploit Snowflake flocculation effectively, increasing their relative fitness. At low densities, these opportunities diminish, limiting Co-Uni persistence despite its higher intrinsic growth rate.

While classic game theory models typically assume well-mixed populations with fixed payoffs, our results highlight how ecological context, in this case: interaction frequency shaped by population density, can shift the payoff landscape itself. This is supported by patterns observed in microbial sucrose metabolism, where cheating by non-invertase producers becomes more successful at higher cell densities [22]. This suggests that density can serve as a critical axis of selection in systems governed by social exploitation.

### Density as a scaffold for microbial social evolution

Microbial cooperation often evolves in environments where interaction frequencies are high enough for public goods to be reliably shared among related or reciprocating cells. In our system, we demonstrate that population density serves as a key ecological parameter modulating these interaction frequencies: only when Snowflake clusters are sufficiently abundant can Co-Uni cells attach and gain access to the settled fraction. This interaction-dependent fitness benefit enables Co-Uni persistence under high-density conditions but breaks down at low density, where encounters are rarer. Thus, social trait coexistence in this system is not simply a product of frequency-dependent selection, but of density-contingent interaction regimes that constrain short-term ecological dynamics and may influence longer-term evolutionary outcomes.

This logic may generalize to more complex microbial systems. Structured environments such as biofilms provide spatial scaffolds that increase local density and promote repeated interactions, reinforcing the benefits of cooperation [2,47–51]. Aggregative multicellular forms like those seen in Snowflake yeast mimic this logic, enabling stabilizing dynamics. Similar principles have been observed in other microbial taxa. In *Myxococcus xanthus*, fruiting body formation requires a critical cell density, below which multicellular development and cooperative feeding fail [52]. Cheater mutants that exploit these processes thrive only when rare and are suppressed under high-density, clonal conditions [53]. Likewise, *Dictyostelium discoideum* exhibits density thresholds for cooperative stalk formation and reduced success of cheating genotypes at low population densities [23,24,54,55]. In both cases, social benefits emerge only when density enables sufficient coordination and enforcement of cooperative behaviors.

These dynamics raise the possibility that density itself can function as a scaffold for social complexity, shaping the evolutionary trajectories of microbial communities. One consequence of sustained high-density interaction is the emergence of metabolic interdependencies, wherein species lose redundant functions and become obligately linked. This phenomenon is formalized in the Black Queen Hypothesis, which posits that public-good producers enable gene loss and specialization among exploiters [56–59]. While our system does not involve direct metabolic exchange, it illustrates how physical associations and interaction-dependent benefits could enable trait loss or asymmetry under stable, high-density conditions. In oligotrophic or resource-limited environments, such density-sensitive architectures may permit cooperation to emerge, persist, or specialize in ways that would be unstable under well-mixed or dilute conditions.

Ultimately, our findings suggest that density-dependent interactions are not merely ecological side effects, they are evolutionary constraints. By controlling the frequency of social encounters, density influences which traits persist, which are lost, and how new cooperative strategies emerge. Understanding these dynamics may help explain the origins of complex social behaviors in simple aggregative microbes and guide predictions about how interdependent communities evolve and stabilize over time.

## Conclusion

Our findings demonstrate that population density plays a decisive role in shaping short-term ecological outcomes in cooperator–cheater interactions in microbial systems, with potential consequences for longer-term evolutionary dynamics. By manipulating total cell density while holding initial strain frequencies constant, we show that the ability of a non-cooperative strain to persist depends critically on the frequency of physical interactions with a cooperative partner. At high densities, Snowflake yeast form aggregative structures that allow Co-Uni cells to exploit settling benefits; at low densities, these interactions become rare, weakening this stabilizing mechanism and reducing Co-Uni persistence. These results offer a direct experimental test of coexistence theory, illustrating how ecological parameters, specifically, interaction frequency mediated by density, govern the balance between stabilizing and equalizing forces.

This work contributes to a growing understanding of how simple ecological variables can modulate complex evolutionary outcomes. While many studies have emphasized genetic or spatial structure in shaping microbial cooperation, our results show that interaction frequency alone, even in the absence of evolved enforcement or structured environments, can determine whether social traits persist or collapse. This highlights density as a powerful and generalizable constraint on the evolution of microbial communities.

Future work could explore how these density-dependent dynamics extend to systems involving metabolic interdependence or long-term coevolution. More broadly, understanding how ecological structure shapes selection for cooperation and exploitation may illuminate the conditions under which multicellularity, division of labor, and obligate symbioses evolve. By identifying interaction frequency as a modifiable ecological axis, our study provides a framework for investigating the early scaffolds of social evolution in microbial populations.

## Supporting information

S1 File S1 Table. Linear mixed-effects model results of the effects of Time, Phenotype and their interactions on the natural log of population density estimates (CFU/ml). **S2 Table.** ANOVA results of the effects of Time, Phenotype and their interactions on the natural log of population density estimates (CFU/ml). **S1 Fig.** Snowflake and Co-Unis co-exist over time and Co-Uni persistence is not driven by adaptive changes. A) Change in population density of Co-Uni and Snowflake strains over 15 transfers in coculture, shown as the natural logarithm of colony forming units per ml (ln[CFU/ml]). Lines represent predicted population densities by the linear mixed-effects model. Transfers were conducted every 24 hours under settling selection. B) Change in population density (ln[CFU/ml]) of co-cultures with Co-Uni isolates from the beginning (Day 0) and end (Day 15) of the experiment over 6 transfers. Lines represent predicted population densities by the linear mixed effects model. Transfers were conducted every 24 hours under settling selection. The minimum detection threshold for both strains was approximately 12.5 ln(CFU/ml). **S3 Table.** Results of linear mixed-effects model analysing the effects of transfer number, phenotype and state (Evolved or ancestral) on population density. **S4 Table.** Type III ANOVA results for linear-mixed effects model testing the effects of transfer number, phenotype, and state on population density. **S5 Table.** Two-way ANOVA results for growth rates of Co-Uni and Snowflake isolates, analyzing the effects of phenotype and transfer number. **S2 Fig.** A) Optical Density measurements (600 nm) for Snowflake and Co-Uni isolates from the beginning (Transfer number 0 in green) and end (Transfer number 15 in purple) of the experiment over time, fitted using a locally estimated scatterplot smoothing (LOESS). B) Intrinsic growth rates for Snowflake and Co-Uni isolates from the beginning (Transfer number 0) and end (Transfer number 15) of the experiment as calculated using the growthcurver package in R. **S6 Table.** Linear mixed-effects model results for population density influenced by time phenotype, and density. **S7 Table.** ANOVA results for effects of time, phenotype, and density on population density. **S8 Table.** Fit parameters and model performance for replicate-level log_10_(Co-Uni CFU / Snowflake CFU) ratio trajectories across settling densities. **S9 Table.** Linear regression results testing the relationship between settling density and Co-Uni selection strength (slope). **S10 Table.** ANOVA results testing the effect of settling density on Co-Uni selection strength (slope). **S11 Table.** Linear regression on asymptote ratios for Co-Uni and Snowflake frequency dependence. **S12 Table.** ANOVA for asymptote ratio predicting Co-Uni and Snowflake equilibrium frequency. **S13 Table.** Parameters from exponential decay fits of Co-Uni CFU at different settling densities. **S14 Table.** Logistic growth parameters for snowflake CFU across settling densities. **S15 Table.** Linear regression analysis of Co-Uni CFU at equilibrium against settling density. **S16 Table.** ANOVA results for Co-Uni CFU predictions at equilibrium across settling densities. **S17 Table.** Linear regression analysis of Co-Uni CFU decay slope against settling density. **S18 Table.** ANOVA results for Co-Uni CFU decay slope across settling densities. **S3 Fig.** Model fits and density-dependent dynamics of Co-Uni CFU trajectories. A) Log_10_-transformed Co-Uni CFU counts over time for each biological replicate, across different relative settling densities. Lines represent replicate-level linear fits to the log-transformed CFU data; points represent raw measurements. B) Relationship between settling density and decay slope estimated from replicate-level fits. More positive slopes at higher densities indicate slower Co-Uni population decline. C) Predicted equilibrium CFU counts (asymptotic values) extracted from exponential decay models, plotted against relative settling density. **S19 Table.** Linear regression analysis of Snowflake CFU at equilibrium against settling density. **S20 Table.** ANOVA results for Snowflake CFU predictions at equilibrium across settling densities. **S21 Table.** Linear regression analysis of Snowflake CFU decay slope against settling density. **S22 Table.** ANOVA results for Snowflake CFU decay slope across settling densities. **S4 Fig.** Model fits and density-dependent dynamics of Snowflake CFU trajectories. A) Log_10_-transformed Snowflake CFU counts over time for each biological replicate, across different relative settling densities. Lines represent replicate-level linear fits to the log-transformed CFU data; points represent raw measurements. B) Relationship between settling density and growth slope estimated from replicate-level fits. Slopes remain stable across densities, indicating limited density dependence in Snowflake population expansion. C) Predicted equilibrium CFU counts (asymptotic values) extracted from logistic growth models, plotted against relative settling density. No significant trend is observed. **S23 Table.** Linear regression of relative fitness against settling density. **S24 Table.** ANOVA results for relative fitness across settling densities. **S25 Table.** Linear model analysis of relative increase by settling density and phenotype. **S26 Table.** ANOVA results for relative increase by settling density and phenotype. **S27 Table.** Comparison of candidate linear models predicting Co-Uni settling fitness as a function of density and Snowflake frequency.
(ZIP)
