# Supplementary material for: Population density constrains coexistence of cooperative and cheating Kluyveromyces lactis through frequency-dependent physical interactions
Source: PLoS One. 2026 Oct 1;21(10):e0359779. doi: 10.1371/journal.pone.0359779 (PMC13630254; doi:10.1371/journal.pone.0359779)
Supplement: Supplementary file 1 [file pone.0359779.s001.zip › SI_bundle_final/S14_table.pdf]

S14 Table. Logistic growth parameters for snowflake CFU across settling densities.

Density	Replicate	L	k	x_0	R^2
0.25	1	4718595	0.0707	0.000	0.198
0.25	2	5061179	0.0339	0.000	0.140
0.25	3	3981990	0.0911	0.000	0.269
0.25	5	4900755	0.0438	0.000	0.163
0.50	1	3068190	0.7900	0.950	0.542
0.50	2	2140000	5.4000	0.003	0.352
0.50	3	2929220	0.6430	0.996	0.434
0.50	4	3070141	0.9310	0.892	0.371
0.50	5	3080362	1.0400	0.798	0.414
0.75	1	3322140	1.6400	0.461	0.539
0.75	2	8336846	0.0982	15.000	0.631
0.75	3	5902289	0.0992	9.270	0.644
0.75	4	7699907	0.0890	15.000	0.539
0.75	5	2852009	3.7200	0.109	0.452
1.00	1	2857113	1.0000	0.675	0.450
1.00	2	2953084	0.8340	0.960	0.421
1.00	3	3345174	0.1500	0.000	0.299
1.00	4	2683330	0.7540	0.942	0.380
