# Supplementary material for: Population density constrains coexistence of cooperative and cheating Kluyveromyces lactis through frequency-dependent physical interactions
Source: PLoS One. 2026 Oct 1;21(10):e0359779. doi: 10.1371/journal.pone.0359779 (PMC13630254; doi:10.1371/journal.pone.0359779)
Supplement: Supplementary file 1 [file pone.0359779.s001.zip › SI_bundle_final/S19_table.pdf]

S19 Table. Linear regression analysis of Snowflake CFU at equilibrium against settling density.

**Term**	**β Coefficient**	**Std. Error**	**t-value**	**p-value**
(Intercept)	4.60E+06	1.04E+06	4.43	< 0.001
Density	-9.83E+05	1.49E+06	-0.662	0.517
