# Supplementary material for: Population density constrains coexistence of cooperative and cheating Kluyveromyces lactis through frequency-dependent physical interactions
Source: PLoS One. 2026 Oct 1;21(10):e0359779. doi: 10.1371/journal.pone.0359779 (PMC13630254; doi:10.1371/journal.pone.0359779)
Supplement: Supplementary file 1 [file pone.0359779.s001.zip › SI_bundle_final/S6_table.pdf]

S6 Table. Linear mixed-effects model results for population density influenced by time, phenotype, and density.

Effect	Estimate	Std. Error	df	t value	Pr(>|t|)
(Intercept)	19.348	0.070	275.576	762.535	0.000
Time	-0.286	0.008	-37.068	1168.677	<2e-16
PhenotypeSnowflake	-4.925	0.098	-50.088	1167.902	<2e-16
Density	-0.001	0.001	-0.766	1167.932	0.444
Time:PhenotypeSnowflake	0.334	0.011	30.610	1167.902	<2e-16
Time:Density	0.002	0.000	19.471	1168.146	<2e-16
PhenotypeSnowflake:Density	-0.002	0.001	-1.185	1167.902	0.236
Time:PhenotypeSnowflake:Density	-0.002	0.000	-12.937	1167.902	<2e-16

Random effects:

Variance (Replicate): 0.00047

Residual variance: 0.153

Number of observations: 1180
Groups: Replicate (5 levels)
REML criterion at convergence: 1222.1
