# Supplementary material for: Population density constrains coexistence of cooperative and cheating Kluyveromyces lactis through frequency-dependent physical interactions
Source: PLoS One. 2026 Oct 1;21(10):e0359779. doi: 10.1371/journal.pone.0359779 (PMC13630254; doi:10.1371/journal.pone.0359779)
Supplement: Supplementary file 1 [file pone.0359779.s001.zip › SI_bundle_final/S27_table.pdf]

S27 Table. Comparison of candidate linear models predicting Co-Uni settling fitness as a function of density and Snowflake frequency.

Model	Adjusted R^2	AIC
**Co-Uni settling fitness ~ Density**	**0.681**	**–30.3**
Co-Uni settling fitness ~ log(Density)	0.602	–25.8
Co-Uni settling fitness ~ Density + Snowflake frequency	0.679	–29.3
Co-Uni settling fitness ~ log(Density) + log(Snowflake freq)	0.593	–24.6
