# Supplementary material for: Population density constrains coexistence of cooperative and cheating Kluyveromyces lactis through frequency-dependent physical interactions
Source: PLoS One. 2026 Oct 1;21(10):e0359779. doi: 10.1371/journal.pone.0359779 (PMC13630254; doi:10.1371/journal.pone.0359779)
Supplement: Supplementary file 1 [file pone.0359779.s001.zip › SI_bundle_final/S15_table.pdf]

***S15 Table. Linear regression analysis of Co-Uni CFU at equilibrium against settling density.***

| Term | β Coefficient | Std. Error | t-value | p-value |
|---|---|---|---|---|
| (Intercept) | -1.90E+08 | 2.85E+07 | -6.69 | < 0.001 |
| Density | 3.13E+08 | 4.16E+07 | 7.54 | < 0.001 |
