# Supplementary material for: Population density constrains coexistence of cooperative and cheating Kluyveromyces lactis through frequency-dependent physical interactions
Source: PLoS One. 2026 Oct 1;21(10):e0359779. doi: 10.1371/journal.pone.0359779 (PMC13630254; doi:10.1371/journal.pone.0359779)
Supplement: Supplementary file 1 [file pone.0359779.s001.zip › SI_bundle_final/S18_table.pdf]

S18 Table: ANOVA results for Co-Uni CFU decay slope across settling densities.

**Source of Variation**	**df**	**SS**	**MS**	**F**	**p-value**
Density	1	0.692	0.692	8.94	0.007
Residuals	18	1.39	0.0775	—	—
