# Supplementary material for: Population density constrains coexistence of cooperative and cheating Kluyveromyces lactis through frequency-dependent physical interactions
Source: PLoS One. 2026 Oct 1;21(10):e0359779. doi: 10.1371/journal.pone.0359779 (PMC13630254; doi:10.1371/journal.pone.0359779)
Supplement: Supplementary file 1 [file pone.0359779.s001.zip › SI_bundle_final/S7_table.pdf]

S7 Table. ANOVA results for effects of time, phenotype, and density on population density.

**Source of Variation**	**SS**	**MS**	**NumDF**	**DenDF**	**F**	**p-value**
Time	73.009	73.009	1.000	1169.372	475.833	<2e-16
Phenotype	384.937	384.937	1.000	1167.902	2508.827	<2e-16
Density	0.790	0.790	1.000	1167.961	5.149	0.023
Time:Phenotype	143.763	143.763	1.000	1167.902	936.975	<2e-16
Time:Density	32.705	32.705	1.000	1168.382	213.157	<2e-16
Phenotype:Density	0.216	0.216	1.000	1167.902	1.405	0.236
Time:Phenotype:Density	25.678	25.678	1.000	1167.902	167.357	<2e-16

.
