# Supplementary material for: Population density constrains coexistence of cooperative and cheating Kluyveromyces lactis through frequency-dependent physical interactions
Source: PLoS One. 2026 Oct 1;21(10):e0359779. doi: 10.1371/journal.pone.0359779 (PMC13630254; doi:10.1371/journal.pone.0359779)
Supplement: Supplementary file 1 [file pone.0359779.s001.zip › SI_bundle_final/S26_table.pdf]

S26 Table. ANOVA results for relative increase by settling density and phenotype.

Source of Variation	df	SS	MS	F	p-value
Density	1	0.264	0.264	8.66	0.006
Phenotype	1	38.702	38.702	1268.225	< 0.001
Density:Phenotype	1	0.220	0.220	7.226	0.011
Residuals	36	1.099	0.030		
