# Supplementary material for: Population density constrains coexistence of cooperative and cheating Kluyveromyces lactis through frequency-dependent physical interactions
Source: PLoS One. 2026 Oct 1;21(10):e0359779. doi: 10.1371/journal.pone.0359779 (PMC13630254; doi:10.1371/journal.pone.0359779)
Supplement: Supplementary file 1 [file pone.0359779.s001.zip › SI_bundle_final/S13_table.pdf]

S13 Table: Parameters from exponential decay fits of Co-Uni CFU at different settling densities.

Density	Replicate	a	b	c	R^2
0.25	1	365453781	0.0763	-128546192	0.886
0.25	2	356185976	0.0791	-121293207	0.914
0.25	3	287147584	0.0966	-74711072	0.862
0.25	4	481483612	0.0400	-274094295	0.803
0.25	5	403652786	0.0740	-146715847	0.906
0.5	1	298876736	0.459	31980305	0.834
0.5	2	270700237	0.434	22763681	0.951
0.5	3	377550873	0.406	21199339	0.862
0.5	4	313182046	0.189	-5330515	0.887
0.5	5	247706807	0.278	22444712	0.855
0.75	1	358843215	0.609	64635103	0.760
0.75	2	328260706	0.752	73381455	0.764
0.75	3	330530488	0.374	55990111	0.834
0.75	4	316568272	0.195	34462816	0.823
0.75	5	383879732	0.248	32632503	0.782
1	1	180200057	1.510	115801210	0.102
1	2	191569343	0.471	99955548	0.496
1	3	227130491	0.506	106013511	0.434
1	4	282815055	0.276	76707218	0.714
