# Supplementary material for: Population density constrains coexistence of cooperative and cheating Kluyveromyces lactis through frequency-dependent physical interactions
Source: PLoS One. 2026 Oct 1;21(10):e0359779. doi: 10.1371/journal.pone.0359779 (PMC13630254; doi:10.1371/journal.pone.0359779)
Supplement: Supplementary file 1 [file pone.0359779.s001.zip › SI_bundle_final/S1_table.pdf]

S1 Table. Linear mixed-effects model results of the effects of Time, Phenotype and their interactions on the natural log of population density estimates (CFU/ml).

**Effect**	**Estimate**	**Std. Error**	**df**	**t value**
(Intercept)	19.201	0.055	296.000	347.820
Time	-0.063	0.006	296.000	-10.380
PhenotypeSnowflake	-5.004	0.078	296.000	-64.090
Time:PhenotypeSnowflake	0.117	0.009	296.000	13.640

Random effects:

Variance (Replicate): 0.0000

Residual variance: 0.1455

Number of observations: 300
Groups: Replicate (5 levels)
REML criterion at convergence: 295.9
