# Supplementary material for: Population density constrains coexistence of cooperative and cheating Kluyveromyces lactis through frequency-dependent physical interactions
Source: PLoS One. 2026 Oct 1;21(10):e0359779. doi: 10.1371/journal.pone.0359779 (PMC13630254; doi:10.1371/journal.pone.0359779)
Supplement: Supplementary file 1 [file pone.0359779.s001.zip › SI_bundle_final/S9_table.pdf]

***S9 Table. Linear regression results testing the relationship between settling density and Co-Uni selection strength (slope).***

| **Variables** | **β Coefficient** | **Std. Error** | **t-value** | **p-value** |
|---|---|---|---|---|
| (Intercept) | -0.144 | 0.005 | -31.5 | < 0.001 |
| Density | 0.088 | 0.007 | 13.4 | < 0.001 |
