# Supplementary material for: Population density constrains coexistence of cooperative and cheating Kluyveromyces lactis through frequency-dependent physical interactions
Source: PLoS One. 2026 Oct 1;21(10):e0359779. doi: 10.1371/journal.pone.0359779 (PMC13630254; doi:10.1371/journal.pone.0359779)
Supplement: Supplementary file 1 [file pone.0359779.s001.zip › SI_bundle_final/S23_table.pdf]

S23 Table. Linear regression of relative fitness against settling density.

Variables	β Coefficient	Std. Error	t-value	p-value
(Intercept)	-2.299	0.056	-40.779	< 0.001
Density	0.531	0.082	6.451	<0.001
