# Supplementary material for: Population density constrains coexistence of cooperative and cheating Kluyveromyces lactis through frequency-dependent physical interactions
Source: PLoS One. 2026 Oct 1;21(10):e0359779. doi: 10.1371/journal.pone.0359779 (PMC13630254; doi:10.1371/journal.pone.0359779)
Supplement: Supplementary file 1 [file pone.0359779.s001.zip › SI_bundle_final/S4_table.pdf]

S4 Table. Type III ANOVA results for linear-mixed effects model testing the effects of transfer number, phenotype, and state on population density.

**Source of Variation**	**SS**	**MS**	**NumDF**	**DenDF**	**F**	**p-value**
Time	5.428	5.428	1.000	288	37.844	0.000
Phenotype	699.174	699.174	1.000	288	4875.039	<2e-16
State	0.109	0.109	1.000	288	0.763	0.383
Time:Phenotype	53.234	53.234	1.000	288	371.178	<2e-16
Time:State	0.062	0.062	1.000	288	0.436	0.510
Phenotype:State	0.219	0.219	1.000	288	1.526	0.218
Time:Phenotype:State	0.139	0.139	1.000	288	0.968	0.326
