# Supplementary material for: Population density constrains coexistence of cooperative and cheating Kluyveromyces lactis through frequency-dependent physical interactions
Source: PLoS One. 2026 Oct 1;21(10):e0359779. doi: 10.1371/journal.pone.0359779 (PMC13630254; doi:10.1371/journal.pone.0359779)
Supplement: Supplementary file 1 [file pone.0359779.s001.zip › SI_bundle_final/S25_table.pdf]

S25 Table. Linear model analysis of relative increase by settling density and phenotype.

**Variables**	**β Coefficient**	**Std. Error**	**t-value**	**p-value**
(Intercept)	0.021	0.096	0.22	0.827
Density	0.556	0.14	3.981	<0.001
PhenotypeSnowflake	2.299	0.135	16.992	< 0.001
Density:PhenotypeSnowflake	-0.531	0.198	-2.688	0.011
