# Supplementary material for: Population density constrains coexistence of cooperative and cheating Kluyveromyces lactis through frequency-dependent physical interactions
Source: PLoS One. 2026 Oct 1;21(10):e0359779. doi: 10.1371/journal.pone.0359779 (PMC13630254; doi:10.1371/journal.pone.0359779)
Supplement: Supplementary file 1 [file pone.0359779.s001.zip › SI_bundle_final/S2_table.pdf]

S2 Table. ANOVA results of the effects of Time, Phenotype and their interactions on the natural log of population density estimates (CFU/ml)

**Source of Variation**	**df**	**SS**	**MS**	**F**	**p-value**
Time	1	0.15	0.15	1.0632	0.303
Phenotype	1	597.43	597.43	4107.469	<2e-16
Time:Phenotype	1	27.07	27.07	186.134	<2e-16
Residuals	296				
