# Supplementary material for: Population density constrains coexistence of cooperative and cheating Kluyveromyces lactis through frequency-dependent physical interactions
Source: PLoS One. 2026 Oct 1;21(10):e0359779. doi: 10.1371/journal.pone.0359779 (PMC13630254; doi:10.1371/journal.pone.0359779)
Supplement: Supplementary file 1 [file pone.0359779.s001.zip › SI_bundle_final/S22_table.pdf]

S22 Table. ANOVA results for Snowflake CFU decay slope across settling densities.

Source	df	SS	MS	F	p-value
Density	1	4.09	4.09	1.24	0.281
Residuals	17	56.0	3.30	—	—
