# Supplementary material for: Population density constrains coexistence of cooperative and cheating Kluyveromyces lactis through frequency-dependent physical interactions
Source: PLoS One. 2026 Oct 1;21(10):e0359779. doi: 10.1371/journal.pone.0359779 (PMC13630254; doi:10.1371/journal.pone.0359779)
Supplement: Supplementary file 1 [file pone.0359779.s001.zip › SI_bundle_final/S8_table.pdf]

S8 Table. Fit parameters and model performance for replicate-level log_{10}(Co-Uni CFU / Snowflake CFU) ratio trajectories across settling densities.

Density	Replicate	a	b	c	R^2
0.25	1	198.039	0.429	3.406	0.912
0.25	2	196.813	0.431	3.704	0.931
0.25	3	202.019	0.431	3.810	0.895
0.25	4	174.947	0.332	1.308	0.895
0.25	5	229.844	0.457	5.162	0.937
0.5	1	343.827	0.880	13.780	0.795
0.5	3	449.859	0.720	9.987	0.804
0.5	4	314.324	0.513	9.967	0.846
0.5	5	268.842	0.659	12.311	0.797
0.75	1	378.913	1.095	22.182	0.709
0.75	2	380.092	1.124	26.340	0.743
0.75	3	311.707	0.622	21.714	0.824
0.75	4	297.880	0.484	23.786	0.809
0.75	5	361.896	0.523	18.797	0.804
1	1	295.390	1.371	41.292	0.296
1	2	318.094	0.895	36.316	0.513
1	3	324.083	0.967	40.855	0.450
1	4	358.925	0.562	33.958	0.649
1	5	375.133	0.701	50.041	0.527
