# Supplementary material for: Population density constrains coexistence of cooperative and cheating Kluyveromyces lactis through frequency-dependent physical interactions
Source: PLoS One. 2026 Oct 1;21(10):e0359779. doi: 10.1371/journal.pone.0359779 (PMC13630254; doi:10.1371/journal.pone.0359779)
Supplement: Supplementary file 1 [file pone.0359779.s001.zip › SI_bundle_final/S17_table.pdf]

S17 Table. Linear regression analysis of Co-Uni CFU decay slope against settling density.

Term	β Coefficient	Std. Error	t-value	p-value
(Intercept)	-0.0505	0.152	-0.331	0.744
Density	0.666	0.223	2.99	0.008
