# Supplementary material for: Population density constrains coexistence of cooperative and cheating Kluyveromyces lactis through frequency-dependent physical interactions
Source: PLoS One. 2026 Oct 1;21(10):e0359779. doi: 10.1371/journal.pone.0359779 (PMC13630254; doi:10.1371/journal.pone.0359779)
Supplement: Supplementary file 1 [file pone.0359779.s001.zip › SI_bundle_final/S5_table.pdf]

***S5 Table. Two-way ANOVA results for growth rates of Co-Uni and Snowflake isolates, analyzing the effects of phenotype and transfer number*.**

**Source of Variation**	**df**	**SS**	**MS**	**F**	**p-value**
Genotype	1	0.0346	0.0346	26.2898	0.0000
Transfer	1	0.0070	0.0070	5.2838	0.0256
Genotype:Transfer	1	0.0009	0.0009	0.6463	0.4251
Residuals	52	0.0684	0.0013		
