# Supplementary material for: Population density constrains coexistence of cooperative and cheating Kluyveromyces lactis through frequency-dependent physical interactions
Source: PLoS One. 2026 Oct 1;21(10):e0359779. doi: 10.1371/journal.pone.0359779 (PMC13630254; doi:10.1371/journal.pone.0359779)
Supplement: Supplementary file 1 [file pone.0359779.s001.zip › SI_bundle_final/S11_table.pdf]

S11 Table. Linear regression on asymptote ratios for Co-Uni and Snowflake frequency dependence.

**Variables**	**β Coefficient**	**Std. Error**	**t-value**	**p-value**
(Intercept)	-10.811	2.431	-4.447	< 0.001
Density	48.677	3.508	13.877	< 0.001
