# Supplementary material for: Population density constrains coexistence of cooperative and cheating Kluyveromyces lactis through frequency-dependent physical interactions
Source: PLoS One. 2026 Oct 1;21(10):e0359779. doi: 10.1371/journal.pone.0359779 (PMC13630254; doi:10.1371/journal.pone.0359779)
Supplement: Supplementary file 1 [file pone.0359779.s001.zip › SI_bundle_final/S3_table.pdf]

S3 Table. Results of linear mixed-effects model analysing the effects of transfer number, phenotype and state (Evolved or ancestral) on population density.

**Effect**	**Estimate**	**Std. Error**	**df**	**t value**	**p-value**
(Intercept)	18.776	0.08	36.020	235.428	<2e-16
Time	-0.23	0.018	288	-12.871	<2e-16
PhenotypeSnowflake	-4.913	0.098	288	-50.245	<2e-16
StateEvolved	-0.146	0.098	288	-1.491	0.137
Time:PhenotypeSnowflake	0.362	0.025	288	14.319	<2e-16
Time:StateEvolved	0.006	0.025	288	0.229	0.819
PhenotypeSnowflake:StateEvolved	0.171	0.138	288	1.235	0.218
Time:PhenotypeSnowflake:StateEvolved	-0.035	0.036	288	-0.984	0.326

Random effects:

Variance (Replicate): 0.008

Residual variance: 0.143

Number of observations: 300
Groups: Replicate (5 levels)
REML criterion at convergence: 309.1
